# Practical implementation of artificial intelligence algorithms in pulmonary auscultation examination

**DOI:** 10.1007/s00431-019-03363-2

**Published:** 2019-03-29

**Authors:** Tomasz Grzywalski, Mateusz Piecuch, Marcin Szajek, Anna Bręborowicz, Honorata Hafke-Dys, Jędrzej Kociński, Anna Pastusiak, Riccardo Belluzzo

**Affiliations:** 1 StethoMe, Winogrady 18A, 61-663 Poznań, Poland; 2 Department of Pediatric Pneumonology, Allergology and Clinical Immunology, K. Jonscher Clinical Hospital, Poznań University of Medical Sciences, Szpitalna 27/33, 60-572 Poznań, Poland; 3 Institute of Acoustics, Faculty of Physics, Adam Mickiewicz University, Poznań, Umultowska 85, 61-614 Poznań, Poland

**Keywords:** Auscultation, Artificial intelligence, Machine learning, Respiratory system, Stethoscope

## Abstract

Lung auscultation is an important part of a physical examination. However, its biggest drawback is its subjectivity. The results depend on the experience and ability of the doctor to perceive and distinguish pathologies in sounds heard via a stethoscope. This paper investigates a new method of automatic sound analysis based on neural networks (NNs), which has been implemented in a system that uses an electronic stethoscope for capturing respiratory sounds. It allows the detection of auscultatory sounds in four classes: wheezes, rhonchi, and fine and coarse crackles. In the blind test, a group of 522 auscultatory sounds from 50 pediatric patients were presented, and the results provided by a group of doctors and an artificial intelligence (AI) algorithm developed by the authors were compared. The gathered data show that machine learning (ML)–based analysis is more efficient in detecting all four types of phenomena, which is reflected in high values of recall (also called as sensitivity) and F1-score.

*Conclusions*: The obtained results suggest that the implementation of automatic sound analysis based on NNs can significantly improve the efficiency of this form of examination, leading to a minimization of the number of errors made in the interpretation of auscultation sounds.

**Table:** 

**What is Known:** • *Auscultation performance of average physician is very low. AI solutions presented in scientific literature are based on small data bases with isolated pathological sounds (which are far from real recordings) and mainly on leave-one-out validation method thus they are not reliable.*	
**What is New:** • *AI learning process was based on thousands of signals from real patients and a reliable description of recordings was based on multiple validation by physicians and acoustician resulting in practical and statistical prove of AI high performance.*

## Background

Auscultation has been considered as an integral part of physical examination since the time of Hippocrates. The stethoscope, introduced by Laennec [2] more than two centuries ago, was one of the first medical instruments which enabled internal body structures and their functioning to be checked.

The stethoscope still remains a tool that can provide potentially valuable clinical information. However, the results of such examinations are strongly subjective and cannot be shared and communicated easily, mostly because of doctors’ experience and perceptual abilities, which leads to differences in the their assessments, depending on their specialization (Hafke et al., submitted for publication). Another important issue is the inconsistent nomenclature of respiratory sounds. This problem is widely recognized [1], but to date, there is still no standardized worldwide classification of the types of phenomena appearing in the respiratory system [10]. There is both a variety of terms used for the same sound by different doctors and different sounds described by the same term. Lung sounds, as defined by Sovijarvi et al. [14], concern all respiratory sounds heard or detected over the chest wall or within the chest, including normal breathing sounds and adventitious sounds. In general, respiratory sound is characterized by a low noise during inspiration, and hardly audible during expiration. The latter is longer than the former [12]. The spectrum of noise of normal respiratory sound (typically 50–2500 Hz) is broader on the trachea (up to 4000 Hz) [11].

Adventitious sounds are abnormalities (pathologies) superimposed on normal breathing sounds. They can be divided into two sub-classes depending on their duration: continuous (stationary) sounds—wheezes, rhonchi, and discontinuous (non-stationary) sounds—fine or coarse crackles.

Wheezes are continuous tonal sounds with a frequency range from less than 100 Hz to more than 1 kHz, and a duration time longer than 80 ms [8]. They are generally recognized correctly and rarely misinterpreted, which makes them probably the most easily recognized pathological sound [7]. However, as Hafke et al. (submitted for publication) proved, in the case of describing previously recorded sounds, doctors have difficulty identifying this kind of pathology depending on breathing phase, i.e., inspiratory wheezes were confused with expiratory wheezes and vice versa.

Rhonchi are continuous, periodic, snoring-like, similar to wheezes, but of lower fundamental frequency (typically below 300 Hz) and duration, typically longer than 100 ms [8]. It is one of the most ambiguous classes of pathological sounds, as it is often considered to be on the boundary between wheezes and crackles (especially of coarse type). Thus, they may be mistaken for them [15]. Although many authors suggested “rhonchus” as a separate category [10], some doctors use the term “low-pitch wheeze” [6]. Due to the fact they have the features of both wheezes and crackles, these phenomena are often differently classified by the respondents. As Hafke et al. proved, this is strongly dependent on the examiner’s experience. Moreover, in the cited research, the advantage of pulmonologists was clearly visible. In their case, the number of correct rhonchi detections was 51.2%, while for other groups, this value did not exceed 30%, which was the lowest result for all the phenomena taken into account.

Finally, crackles are short, explosive sounds of a non-tonal character. They tend to appear both during inspiration and expiration. Two categories of this phenomenon have been described—fine and coarse crackles. They vary in typical length (ca. 5 ms and ca. 15 ms, respectively) and frequency (broad-band) and may appear in different respiratory system disorders [3]. This is why the proper detection and evaluation of crackles is of high importance.

Auscultation includes the evaluation of sound character, its intensity, frequency, and pathological signals occurring in the breathing sound. Its subjective nature is widely recognized, which has led to a new era of developments, for instance computer-based techniques.

Recordings made with electronic stethoscopes may be further analyzed by a digital system in terms of its acoustic features and, after proper signal processing, delivered to the doctor at an enhanced level of quality or even complemented by a visual representation, e.g., a spectrogram. The latter should be considered as an association between an acoustical signal and its visual representation, and is beneficial to the learning and understanding of those sounds, not only for medical students [13], but also when it comes to doctors diagnosing patients.

Currently, the subject of the greatest attention in the field of computer-based medicine are neural networks (NNs). NNs are a particularly fast developing area of machine learning which learn from examples, as human do. A decade ago NNs were one of many available classifiers. They were trained on a small set of high-level features and produced probability scores of a sample belonging to one of several predefined classes. Their popularity sharply rose when it was proven that deeper neuron structures are able to learn intermediate features from low-level representations by themselves. These intermediate features learned by the NN are much more distinctive and descriptive in comparison to hand-crafted features in many artificial intelligence (AI) tasks, including audio signal analysis and medicine.

Contemporary deep neural networks (DNNs) operate on raw signals directly and are therefore able to identify and exploit all important dependencies that they provide. But in order to be able to do that, a large number of training examples need to be provided. Yet, after these initial requirements are met, the NN algorithm is able to match or even surpass human performance. This is also believed to be the best strategy for dealing with respiratory sounds.

Therefore, the aim of this study was to compare the efficiency of AI and a group of five physicians in terms of respiratory sounds identification in four main classes of pathological signals, according to [10]: wheezes (with no differentiation to sub-classes), rhonchi, and coarse and fine crackles.

## Material and methods

### Auscultation recordings

The auscultation recording files were gathered from 50 visits performed by pediatricians using StethoMe® and Littmann 3200 electronic stethoscopes. All the recordings were made in the Department of Paediatric Pulmonology (Karol Jonscher University Hospital in Poznan, Poland). The subjects were chosen on random from the patients of the abovementioned hospital. The whole procedure of signal collection (recordings) took 6 months. In this period, patients with different diseases (thus, different pathological sounds) were hospitalized. The decision about the recording was made after auscultation by a pulmonologist working at the hospital.

**Fig. 1:**
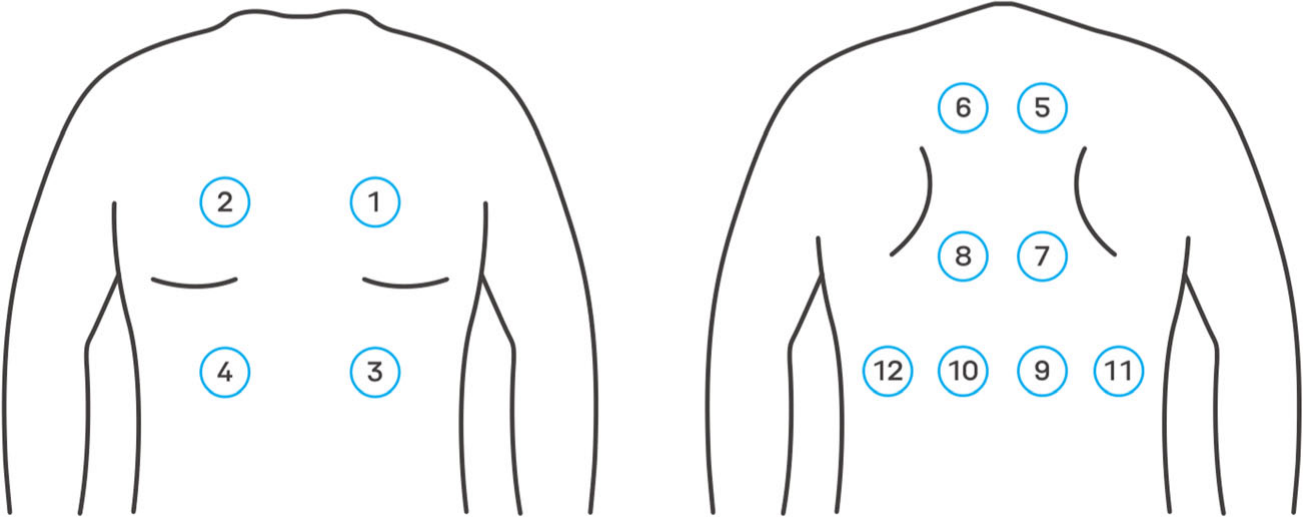
The specific localization of auscultation points in the front (left panel) and back (right panel) of a chest

In general, each visit provided a set of 12 recordings—each of them was made at a different auscultation point (Fig. 1). However, in case of children, as was the case in this research, it is often difficult to document breathing sounds from such a number of auscultation points of a sufficiently high quality, due to children’s movements and impatience, and crying, or because of other health issues. The age of patients was within the range of 1 to 18 years old (mean 8.5; median, 8). This parameter however was not taken into account by AI, but the physicians were informed about the age of each patient. Therefore, the total number of recordings that were analyzed from 50 visits was 522.

### Study design

The main goal was to investigate the accuracy of NNs in the classification of respiratory sounds in comparison with medical specialists. It must be emphasized that, in opposition to most research in many scientific journals which was performed on a small database or in laboratory conditions (e.g., 5, 9), this research was based on a large amount of actual auscultation recordings captured in realistic conditions (hospital). The four abovementioned classes of auscultation phenomena (wheezes, rhonchi, and coarse and fine crackles) were chosen as the most frequently occurring and described. The nomenclature suggested by the European Respiratory Society [10] was applied in order to reduce the influence of ambiguous terminology on the final result. Audio data gathered by electronic stethoscopes was described by doctors in terms of the presence of pathological sounds in certain phases of the breathing cycle and locations on the chest wall. The same description was carried out by the NN.

It should be stressed that together with the recording presentation, the information about the location of the point on the chest or back in which recording was made, as well as basic information about the sex and age of the child, the diagnosis, and accompanying diseases, were provided with every recording of a particular visit. The medical description consisted of the assessment of whether in a given recording, coming from a particular point, there were adventitious respiratory sounds from each of the four classes. Those descriptions were compared both with the NN descriptions as well as with the golden standard (GS).

#### Golden standard

**Fig. 2:**
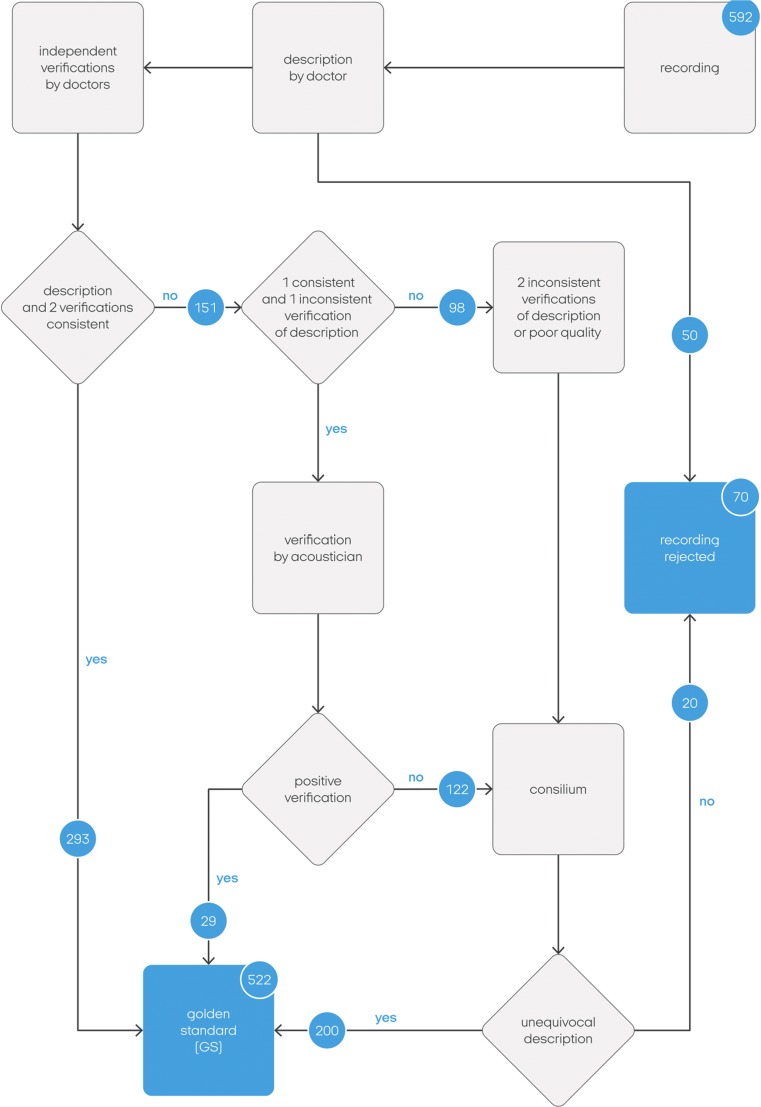
Scheme of the GS data acquisition procedure

Because of the fact that there is no objective measure that provides a classification of pathological breath sounds, it was necessary to establish a point of reference, which in this research is specified as the GS. The mentioned procedure for the GS is depicted in a few steps (Fig. 2.).

Five pediatricians (different from the previous ones) carried out two self-reliant and independent verifications of the previously described recordings. Thus, each recording had a description and two independent verifications. The recordings with double positive medical verifications were automatically qualified to the GS. When the doctors’ opinions were ambiguous—which means there was one positive verification and one negative, the recording was analyzed by an acoustician experienced in signal recognition. Once the acoustician evaluated the description as disputable, which meant its content could be ambiguous in terms of the acoustic parameters, the recording was forwarded to a consilium (2 experienced pediatricians and one acoustician), which was convened to establish a medical description again. It must be emphasized that the GS consisted of real-life recordings collected from real patients in real situations (hospital). Many of the recordings contained additional external noise (crying, talking, stethoscope movements, etc.). To make the GS as reliable as possible, the consilium instead of one physicians described those cases. The descriptions from the consilium were not subjected to further verification (Fig. 2).

Finally, the GS contained 322 recordings with double-positive verification and 200 evaluated by the consilium (Table 1).

**Table 1:** Number of recordings in terms of the appearance of specific pathological phenomena

Phenomenon	Number of recordings
Wheezes	124
Rhonchi	113
Coarse crackles	66
Fine crackles	112

Both no pathology and more than one pathology in one recording were possible; thus, the number of recordings in the Table 1 is not equal to the total number of 522 recordings used in the experiment.

## Participants

### Doctors

The set of all the GS recordings set, accompanied with spectrograms and basic information about each patient, was presented to five pediatricians, and they described them in terms of the occurrence of four pathological sounds (Table 1). One description was made for each recording.

#### NN

**Fig. 3:**
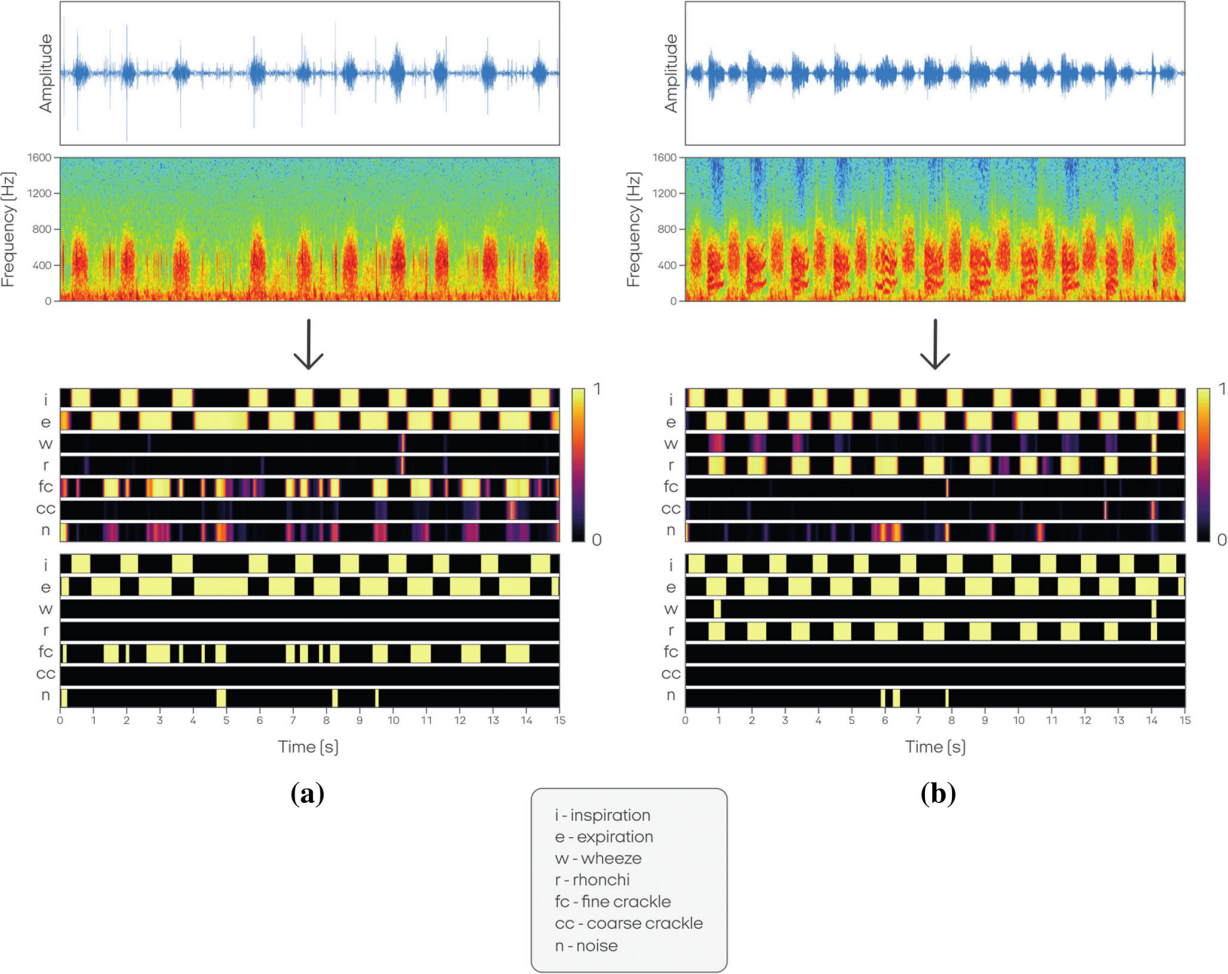
Exemplary probability raster for fine crackles (**a**) and rhonchi (**b**): the signal (first line) is transformed into a spectrogram (second line) and analyzed by the NN. The output of the NN is presented as bidimensional matrix, called a probability raster (third line). The rows in the matrix represent time, framed in windows of 10 ms each; the columns show the probability of positive detection of each phenomenon. The raster is eventually post-processed to obtain boolean values indicating the presence or absence of phenomena for each frame (the fourth line)

StethoMe AI NN architecture based on a modified version of that proposed by Çakir et al. [4] was used. This is a specialized network suitable for polyphonic sound event detection. It is composed of many specialized layers of neurons, including convolutional layers, which are effective at detecting local correlations in the signal, as well as recurrent layers designed to capture long-time dependencies, e.g., a patient’s breathing cycle and the associated recurrence of pathological sounds. The NN had been trained and validated on a set of more than 6000 real and 10,071 artificial/synthetic recordings. This dataset was completely different from the GS set. Furthermore, another database was used in order to provide better noise detection. As output, the NN provided a matrix called the probability raster. In this data structure, the rows represent time, discretized into 10 ms frames, while the columns depict the probability of phenomena detection changing over the frames. The probability values are then thresholded in order to obtain boolean values indicating the presence or absence of such phenomenon along each frame (Fig. 3).

## Analysis

### Results

A GS was used as a point of reference (100%) for tagging recordings performed by doctors and the NN. Therefore, confusion matrices could be analyzed—the values of recall (the proportion of actual positives that are correctly identified as such, also called as sensitivity), precision (the fraction of relevant instances among the retrieved instances), specificity (the proportion of actual negatives that are correctly identified), and the F1-score (the harmonic mean of precision and recall) were measured for the doctors and NN’s phenomena detection in comparison with the GS. First the chi-square test (*α* = 0.05) was performed to investigate if there is a difference in the data gathered for doctors and the NN. The proposed null hypothesis was rejected for all four phenomena. Therefore, the results gathered for the doctors and the NN are statistically different. Detailed results are depicted in Table 2.

**Table 2:** Juxtaposition of recall (sensitivity), precision, specificity, and F1-score for doctors (pediatricians) and NN

	Recall (sensitivity), %		Precision (%)		Specificity (%)		F1-score (%)	
	Doctors	NN	Doctors	NN	Doctors	NN	Doctors	NN
Coarse crackles	56.1	56.1	34.6	40.7	84.6	88.2	42.8	47.1
Fine crackles	72.3	83.9	39.5	52.5	69.8	79.3	51.1	64.6
Wheezes	58.1	78.2	66.1	57.7	90.7	82.2	61.8	66.4
Rhonchi	67.3	87.6	55.9	61.1	85.3	84.6	61.0	72.0
Mean	63.5	76.5	49.0	53.0	82.6	83.6	54.2	62.5

The lowest F1-score was observed for coarse crackles both in the case of medical and NN descriptions. This may be partially due to the rare occurrence of coarse crackles in the analyzed database (see Table 1). Moreover, this kind of phenomena is often confused with other types of crackles or rhonchi (Hafke et al., submitted for publication) so its correct detection might be problematic. However, it is important to note that the NN F1-score which is related to its performance in correct phenomena detection is higher than in the case of medical descriptions (47.1% vs. 42.8%).

The highest F1-score was obtained for rhonchi and wheezes (both continuous, “musical” sounds). Medical descriptions for rhonchi are comparable to the GS (which is reflected in F1-score value) in 61.0%, while NN is much more accurate—72.0%. This is undeniable proof of the ambiguous character of rhonchi, which results in poor detection performance (probably caused by mistaking them for other phenomena, as evidenced by low precision and recall (sensitivity) values when compared to the NN).

When it comes to wheezes, despite the slightly lower values of precision and specificity noted for the NN, its final performance, expressed in F1-score value, is better than in the case of human tagging. The results are as follows—61.8% and 66.4%, with NN superiority.

It can also be noted that the AI-based analysis is more accurate in detecting rhonchi and wheezes. This may be due to the fact that it is based mainly on the spectrograms, which accurately reflect tonal content in a recording. For the doctors, descriptions are mainly based mainly on acoustical cues, while the visual representation is used rather as an additional, supporting tool. This may be an important issue influencing the proper detection of pathology, especially when phenomena is of ambiguous nature (e.g., rhonchi) or accompanied by louder sounds, which make them barely audible (e.g., silent wheezes).

The biggest differences in F1-scores, meaning a significant predominance of the new automatic system over doctors, are observed for fine crackles—64.6% vs. 51.1%. Also, all of other parameters are higher for the NN.

Generally, for each of the four phenomena, the F1-score for the NN is higher than for doctors with an average of 8.4 percentage points (p.p.), which clearly indicates the advantage of the tested algorithm over the group of doctors. NN is 13 p.p. in average more sensitive and 4 p.p. more precise than the reference group of pediatricians.

### Discussion

The main goal of this research was to investigate the effectiveness of pathological respiratory sounds detection for both doctors and the automatic analyzing system based on the NNs developed by the authors.

To measure the performances, the GS was established as a set of 522 recordings taken from the respiratory system of 50 pediatric patients and gathered during auscultation using electronic stethoscopes in real situations. Since auscultation tends to be subjective and there is not an objective measure of correctness, those recordings were then tagged (described) by doctors and experienced acousticians in terms of pathological phenomena content. The recordings with consistent taggings were taken as a point of reference. The inconsistent ones were described by a consilium (2 experienced pediatricians and one acoustician). Only positively verified recordings were used in the next steps of the experiment. In this way, a very reliable GS was established which was taken as a point of reference for the evaluation and comparison of the descriptions of both doctors and the newly developed NN. Since the statistical analysis showed that the performance of those two groups (the doctors and NN) are significantly different, it is reasonable to state that that ML-based analysis that uses the NN algorithm introduced here is more efficient in detecting all four pathological phenomena (wheezes, rhonchi, and coarse and fine crackles), which is reflected in the high values of recall (sensitivity) and the F1-score. It is worth noting that the biggest difference between the performance of doctors and the NN was observed in the case of coarse crackles, where the NN clearly outperformed. Moreover, it has to be mentioned that the NN performance is also higher than that of the doctors in the case of ambiguous sounds (i.e., rhonchi) which tend to be misinterpreted or evaluated in an improper way in everyday medical practice. Finally, the difference between the performance of the doctors and the NN was less significant when it came to the recognition of wheezes; however, this is just because the performance of doctors with those signals which are easiest to interpret is relatively high. Thus, the potential of the proposed solution seems to be enormous. It must be also emphasized that the NN algorithm was taught using thousands of recordings and taggings, which makes the results unique and reliable.

## Conclusions

To conclude, the NN algorithms that were used in this experiment can be described as a very efficient tool for pathological sound detection. This is why AI may become a valuable support for doctors, medical students, or care providers (also lay ones), both when it comes to diagnosing or monitoring processes, on the one hand, and training or education on the other. The database we built is itself a very good tool in this field. Moreover, the AI algorithms can be also beneficial for lay people in terms of monitoring their respiratory system at home, which makes this solution valuable in many areas, e.g., patient safety; reaction speed in case of danger; and, for reducing, the cost of treatment.

It also must be emphasized that there are many publications that correlate pathological sounds with particular disease; however, it is more complicated. There are many publications that show that efficiency of physicians is very low [1, 10]; thus, the AI solution is a first step in making auscultation more objective with less incorrect identification and thus better correlation with diseases made by physicians.

Finally, AI algorithms can also be used in other areas, such as heart disease, which makes this area even more promising, especially taking into account that the results from this experiment which was carried out in real conditions, not in a laboratory with proven high performance of NN.

## Abbreviations

AI: Artificial intelligence
DNN: Deep neural networks
GS: Golden standard
ML: Machine learning
NN: Neural networks;
